# Pyruvate produced by *Brugia* spp. via glycolysis is essential for maintaining the mutualistic association between the parasite and its endosymbiont, *Wolbachia*

**DOI:** 10.1371/journal.ppat.1008085

**Published:** 2019-09-30

**Authors:** Denis Voronin, Emily Schnall, Alexandra Grote, Shabnam Jawahar, Waleed Ali, Thomas R. Unnasch, Elodie Ghedin, Sara Lustigman

**Affiliations:** 1 Molecular Parasitology, New York Blood Center, New York, New York, United States of America; 2 Center for Genomics and Systems Biology, Department of Biology, New York University, New York, New York, United States of America; 3 Center for Global Health Infectious Disease Research, University of South Florida, College of Public Health, Tampa, Florida, United States of America; 4 College of Global Public Health, New York University, New York, New York, United States of America; Pennsylvania State University, UNITED STATES

## Abstract

Human parasitic nematodes are the causative agents of lymphatic filariasis (elephantiasis) and onchocerciasis (river blindness), diseases that are endemic to more than 80 countries and that consistently rank in the top ten for the highest number of years lived with disability. These filarial nematodes have evolved an obligate mutualistic association with an intracellular bacterium, *Wolbachia*, a symbiont that is essential for the successful development, reproduction, and survival of adult filarial worms. Elimination of the bacteria causes adult worms to die, making *Wolbachia* a primary target for developing new interventional tools to combat filariases. To further explore *Wolbachia* as a promising indirect macrofilaricidal drug target, the essential cellular processes that define the symbiotic *Wolbachia*-host interactions need to be identified. Genomic analyses revealed that while filarial nematodes encode all the enzymes necessary for glycolysis, *Wolbachia* does not encode the genes for three glycolytic enzymes: hexokinase, 6-phosphofructokinase, and pyruvate kinase. These enzymes are necessary for converting glucose into pyruvate. *Wolbachia*, however, has the full complement of genes required for gluconeogenesis starting with pyruvate, and for energy metabolism via the tricarboxylic acid cycle. Therefore, we hypothesized that *Wolbachia* might depend on host glycolysis to maintain a mutualistic association with their parasitic host. We did conditional experiments *in vitro* that confirmed that glycolysis and its end-product, pyruvate, sustain this symbiotic relationship. Analysis of alternative sources of pyruvate within the worm indicated that the filarial lactate dehydrogenase could also regulate the local intracellular concentration of pyruvate in proximity to *Wolbachia* and thus help control bacterial growth via molecular interactions with the bacteria. Lastly, we have shown that the parasite’s pyruvate kinase, the enzyme that performs the last step in glycolysis, could be a potential novel anti-filarial drug target. Establishing that glycolysis is an essential component of symbiosis in filarial worms could have a broader impact on research focused on other intracellular bacteria-host interactions where the role of glycolysis in supporting intracellular survival of bacteria has been reported.

## Author summary

*Wuchereria bancrofti*, *Brugia malayi*, *Brugia timori*, and *Onchocerca volvulus* are human parasitic nematodes and causative agents of lymphatic filariasis (LF, elephantiasis) and onchocerciasis (river blindness). These neglected tropical diseases are ranked in the top ten for the highest years lived with disability. In 2016, the global prevalence of LF was between 24.8 to 36.1 million while for onchocerciasis it was 9.5 to 24.2 million. These nematodes have evolved a mutualistic association with an intracellular bacterium, *Wolbachia*, an obligatory symbiont that is essential for the successful development, reproduction, and survival of adult filarial worms. Consequently, these bacteria are a target of interest for developing new interventional tools to combat the filariases. To develop macrofilaricidal drug targets, we must identify the cellular processes that define the symbiotic relationship between the bacteria and their filarial hosts. Here, we evaluated the role of glycolysis—a pathway that is incomplete in *Wolbachia*—in the symbiotic relationship. We found that glycolysis is an essential metabolic pathway that supports the symbiosis between the filarial worm and *Wolbachia*, and demonstrated that this pathway, on which *Wolbachia* rely, can be exploited to discover new or repurposed drugs, and thus facilitate anti-filarial elimination goals.

## Introduction

Human parasitic nematodes such as *Wuchereria bancrofti*, *Brugia malayi*, *B*. *timori*, and *Onchocerca volvulus* are responsible for lymphatic filariasis (LF, elephantiasis) and onchocerciasis (river blindness) in more than 80 countries. In 2016, the global prevalence of LF was in the range of 24.8 to 36.1 million while for onchocerciasis it was 9.5 to 24.2 million [1]. These neglected tropical diseases ranked in the top ten for the highest years lived with disability [1]. Current control measures depend on annual mass drug administration (MDA) programs that are focused on blocking the transmission of infection with the use of drugs that kill microfilariae (albendazole and ivermectin or diethylcarbamazine citrate for LF; and ivermectin only for onchocerciasis). These are unfortunately not macrofilaricidal, i.e. they are not effective against the adult worms. As adult filarial worms live for 6–20 years, MDA requires long term regimens—8 years for LF and 15–20 years for onchocerciasis [2]. Moreover, MDA with ivermectin is hindered by 3 major barriers: a) MDA cannot be deployed in regions co-endemic with *Loa loa* with a high prevalence of microfilaremia because of the risk of severe adverse reactions [3,4]; b) the emergence of drug resistance in filarial nematodes threatens to prolong transmission, despite annual rounds of MDA [5]; and c) current MDA cannot be administered to children under 5 years of age or pregnant women [6]. Therefore, to accelerate elimination there is a critical need for safe and affordable drugs with macrofilaricidal effects. To facilitate these efforts more basic studies that focus on understanding the biology of these parasites at the molecular level are required.

Filarial nematodes that cause LF or river blindness have evolved a mutualistic association with an intracellular bacterium, *Wolbachia* [7,8]. *Wolbachia* is an obligatory symbiont and essential for the successful development, reproduction, and survival of adult filarial worms [9]. Consequently, these bacteria became a target of interest for developing new interventional tools to combat the filariases. A six-week course of doxycycline was demonstrated to clear *Wolbachia*, leading to the sterilization of 80–90% of female worms by 20 months after treatment [10–12], and to a slow death of the adult worms. Unfortunately, antibiotic (doxycycline) treatment is not logistically viable as a MDA strategy due to the recommended regimen requiring high doses over an extended period of time [10,11,13,14]. The use of doxycycline as a macrofilaricidal is therefore recommended only as an alternative option to treat individual patients, or as a ‘test-and-treat’ strategy [15,16]. Studies have also been initiated to obtain safer and faster acting antibiotics that belong to the tetracycline class of drugs [17].

To further explore *Wolbachia* as an indirect macrofilaricidal drug target, we need to better understand the cellular and molecular processes that define the symbiotic relationship with their filarial host. Genomic analyses reveal that while the filarial nematodes encode all the enzymes necessary for glycolysis, *Wolbachia* does not encode the genes for three glycolytic enzymes that are necessary for converting glucose into pyruvate: hexokinase, 6-phosphofructokinase, and pyruvate kinase [18–21]. Pyruvate is one of the most essential metabolites in prokaryotic cells. Notably, *Wolbachia* has the full complement of genes that can use pyruvate for gluconeogenesis and for energy metabolism via the tricarboxylic acid cycle (TCA cycle).

In our previous studies, we hypothesized that glycolysis performed by the filarial worms provides pyruvate to the bacteria, indicating that this pathway plays an important role in maintaining the mutualistic association [21]. We demonstrated that surface protein wBm00432 of the *Wolbachia* (*w*Bm) of *B*. *malayi* was associated with six glycolytic enzymes: fructose-1,6-bisphosphate aldolase, triosephosphate isomerase, lactate dehydrogenase, enolase, glyceraldehyde-3-phosphate dehydrogenase, and phosphoglycerate kinase [20]. Moreover, we have shown that aldolase was important for the survival of both the *Wolbachia* and its filarial host, as well as for reproduction in the female worm [21]. In the current study, we describe how glycolysis and its end-product, pyruvate, contribute to the symbiotic relationship. Using a set of conditional *in vitro* experiments, we show that pyruvate is essential for bacterial growth and, consequently, for parasite reproduction and viability. We also evaluated pyruvate kinase as a potential drug target for anti-filarial treatment. This enzyme performs the last reaction of the glycolytic pathway in the filarial host, a step that is not encoded by the *Wolbachia* genome and for which the bacteria cannot compensate. We also evaluated alternative sources of pyruvate within the worm and show that the filarial lactate dehydrogenase (LDH), an enzyme that catalyzes the conversion between lactate and pyruvate, is colocalized with *Wolbachia*. We predict that LDH could regulate the local intracellular concentration of the host pyruvate in proximity to *Wolbachia* and thus help control bacterial growth, as well as other molecular interactions between the bacteria and their filarial host.

## Materials and methods

### Ethics statement

All animal work conducted by the NUH/NIAID Filariasis Research Reagent Resource Center (FR3) followed the national and international guidelines outlined by the National Institutes of Health Office of Laboratory Animal Welfare and was approved by the University of Georgia Athens and the University of Wisconsin-Oshkosh Institutional Animal Care and Use Committees under protocol numbers A2013 11–009 and 0026-000229-R1-04-25-13, respectively.

### Parasite material and treatments

*B*. *pahangi* and *B*. *malayi* parasites recovered from the peritoneal cavity of infected gerbils (*Meriones unguiculatus*) were obtained from FR3, University of Georgia, Athens. Adult *Acanthocheilenema viteae* parasites recovered from infected Golden Syrian LVG Hamsters were provided by FR3, University of Wisconsin-Oshkosh (WI, USA). The *B*. *pahangi* genome and life cycle are very similar to that of *B*. *malayi* [22], and both species can be maintained in gerbils, although *B*. *pahangi* worm recovery is more efficient in the natural host. This motivated us to use *B*. *pahangi* worms as a substitute species for *B*. *malayi*, and to use *B*. *malayi* to validate specific data when needed.

Adult *B*. *pahangi* worms (2–3 worms) were placed in 3 ml of complete culture medium (RPMI-1640 supplemented with 10% FBS, 100 U/mL penicillin, 100 mg/mL streptomycin, 2 mM L-glutamine) and incubated at 37°C under 5% CO_2_ conditions. Worms were treated with the following compounds: sodium pyruvate (12.5 mM [23]), 3-BromoPyruvate (inhibitor of glycolysis, 3BrPyr, 15 μM), Sodium oximate (SO, 10 mM), sodium lactate (10 mM), or Pyruvate kinase inhibitor III (PKI-III, 12.5 μM). Control worms were cultured in media supplemented with DMSO (0.1%). Media supplemented with the various compounds or DMSO were changed every second day.

### Quantification of microfilariae released by female worms and viability test

As the presence of *Wolbachia* and treatments aimed at the fitness of the symbiont may affect embryogenesis of the adult female worms, we measured the number of microfilariae (Mf) that were released from the adult females during the last two days (4–6 days) of treatment. On the 4^th^ day of treatment, worms were moved into wells containing fresh control or conditional media, and on the 6^th^ day three 20 μl aliquots were taken from each well for counting the total number of Mf using a stereoscope (Olympus). Each treatment was repeated 4 or 5 times as biological replicates. Significance was determined using an unpaired t-test.

Viability of worms was determined using the MTT (3-(4,4-dinethylthiazol-2-yl)-2,5-diphenyltetrazolium bromide) assay [21] after 6 days of treatment and in control parasites. The control and treated adult parasites (*B*. *pahangi* and *A*. *viteae*) were washed with PBS first and then incubated with MTT (0.5 mg/ml) at 37°C under 5% CO_2_ conditions for 1 hour, followed by an additional wash of the parasites with PBS and the addition of 100% DMSO. The DMSO soluble supernatant of five individual worms (treated or control) were transferred into a 96-well plate and the amount of formazan present was measured by Optical Density at 490 nm. The OD that corresponded to control worms was used as a measure of 100% viability of the worms. Significance was determined using an unpaired t-test.

### Concentration of pyruvate within worms

The concentration of pyruvate within the treated worms was analyzed using the Pyruvate Assay kit (Sigma) following the manufacturer’s protocol for tissue samples. Two adult *B*. *pahangi* females per biological sample and 5 biological repeats per experimental group were used in all analyses. Significance was determined using an unpaired t-test.

### Quantification of *Wolbachia*

DNA was extracted from adult *B*. *pahangi* male worms collected after a 6-day treatment using the QIAGene DNA extraction kit (QIAGEN) following manufacturer instructions. The number of *Wolbachia* within each worm was quantified by qPCR using *wsp*, a single-copy gene of *Wolbachia*, as previously described [21,24,25]. Significance was determined using an unpaired t-test.

### LDH enzymatic assay

Soluble crude extracts prepared from adult *B*. *malayi* or *A*. *viteae* worms were used for the LDH enzymatic assay following the manufacturer’s recommendations for the LDH assay kit (Sigma). Two adult females per sample and three biological replicates were used in the assay. Human LDH (provided within the kit) was used as a control for the reaction.

### Gene expression

Total RNA was extracted from 2 female adult *B*. *malayi* worms for each sample using a TRIzol-based method and PureLink RNA Mini Kits with on-column DNase I treatment (Ambion), with 5 biological replicates per experimental group. cDNA synthesis was done using the SuperScript III First Strand cDNA Synthesis Kit (Invitrogen) following the manufacturer’s protocol. Expression levels of the *B*. *malayi LHD* gene (Bm3339), and *Wolbachia* wBm0209 and wBm0207 genes were estimated using the standard ‘ΔΔCt’ method using primers designed with the Primer Premier 4.0 program. The expression of the *B*. *malayi* histone H3 (Bm12920) [26] or *Wolbachia wsp* (wBm0432) genes were used as internal controls (reference genes) [27]. Ct values for each biological replicate were generated using three technical replicates. All data were analyzed using GraphPad Prism 6 and significance was determined using an unpaired t-test.

### Protein expression and pull-down assay

Production of the recombinant protein *w*Bm0432 (*Wolbachia* surface protein) containing a 6xHis tag on the N-terminal was done as previously described [20]. To express *Bm-LDH*, the cDNA of the *Bm*-*LDH* gene (Bm3339) was amplified first by PCR from adult *B*. *malayi* cDNA using a gene-specific primer set: forward-5-CACCAGCTGCGATCGCCTTTGCTTTTCTAGGGAGACT and reverse-5-AACTGTTTAAACAAGACCTTTGATACCGCATT. The first four nucleotides in the forward primer, CACC, were needed for subsequent recombination into the destination vectors. The PCR product was then cloned into pENTR/D-TOPO, the entry cloning vector of the Gateway System (Invitrogen, USA). The cloned cDNA was transferred between the specific attachment sites (attL and attR) on the entry clone and the pDEST15 destination vector using the Gateway LR Clonase Plus enzyme mix (LR recombination) following the manufacturer’s instructions (Invitrogen). This LR recombination provided a GST Tag at the N-terminal end of the expressed Bm-LDH protein. The LDH-Bm3339-pDEST15 plasmid was transformed into BL21 *Escherichia coli*. Single colonies of *E*. *coli* transfected with Bm-LDH and controls without Bm-LDH were cultured in 5 ml LB broth for 16h (overnight) at 37°C. A calculated amount of overnight culture was inoculated in 50 ml LB broth for a final OD_600_ of 0.05 and incubated at 30°C while shaking, periodically measuring the concentration until the OD_600_ reached 0.1. IPTG (Sigma) was then added at a final concentration of 0.4 mM and the cultures were incubated at 30°C for another 2h. Aliquots of cell cultures (2 ml) were then pelleted and stored at -80°C until use.

The Pierce GST Protein Interaction Pull-Down Kit (ThermoFisher) was used to distinguish complexes formed between *w*Bm0432-HIS and Bm-LDH-GST proteins, following the manufacturer’s instructions. Column 1 had *w*Bm0432-HIS [20] immobilized on Ni-NTA beads (TheromoFisher) that were incubated with control crude bacterial extract (bacteria that did not express recombinant LDH). Column 2 had free Ni-NTA beads that were incubated with control crude bacterial extract. Column 3 had *w*Bm0432-HIS immobilized on Ni-NTA beads that were incubated with crude bacterial extract expressing Bm-LDH-GST. Finally, column 4 had free Ni-NTA beads that were incubated with bacterial extract expressing Bm-LDH-GST. A Western blot was done with the pull-down assay products using anti-GST antibody conjugated with HRP (1:200) (Sigma) and Anti-6X His-HRP antibody (1:2000) (AbCam).

### Microscopy

To study the localization of Bm-LDH using confocal microscopy, *B*. *malayi* adult females were fixed in 4% formaldehyde in PBS containing 0.01% Triton-X100 (PBS-T) for 20 min. During fixation, worms were cut into small fragments to improve diffusion of the antibodies into worm tissue. Samples were washed three times in PBS-T, blocked with 5% BSA for 15 min, and then incubated overnight at 4°C with rabbit anti-human LDH antibody (Anti-LDHA, Cat: SAB2108638, Sigma) diluted 1:200 in blocking solution. The secondary anti-rabbit antibody labelled with FITC was used at 1:250. After incubation with the antibodies, samples were co-stained with DAPI, which was added to the mounting medium (Vectashield) to visualize DNA (host nuclei and *Wolbachia*). Stained samples were viewed with an LSM 880 confocal microscope (Zeiss).

### Evaluation of stage-specific expression of *Brugia* genes using transcriptomic data

All *B*. *malayi* transcriptomic data were obtained from the NCBI Short Read Archive database (SRP090644). L3 samples (3 biological replicates) were collected from infected mosquitoes. L4 and adult worms were collected from infected animals (*in vivo* model) and supplied by FR3 [27]. Data obtained for the transcriptomic analysis of L4 samples consisted of data collected from L4 at days 9 and 14 post infection providing 4 biological replicates. The data obtained for the analysis of transcripts of adult (male or female) worms were combined from samples collected on days 42 and 120 post infection with 4 biological replicates for each sex. FPKM (Fragments Per Kilobase Per Million Mapped Fragments) was used to calculate fold change (FC) by dividing FPKM of a gene of interest (GOI) in L4, adult male, or adult female worm samples by FPKM of the same GOI in the L3 samples. Log_2_FC was used to present the results. The data were analyzed using GraphPad Prism 6 and significance between L4 vs L3 or adult (male or female) vs L3 was determined using an unpaired t-test. EdgeR [28] was also used to calculate adjusted CPMs (Counts Per Million), normalizing for library size between samples; we observed a similar expression profile using this second normalization method.

## Results

### External pyruvate facilitates *Wolbachia* growth in *Brugia* parasites

**Fig 1:**
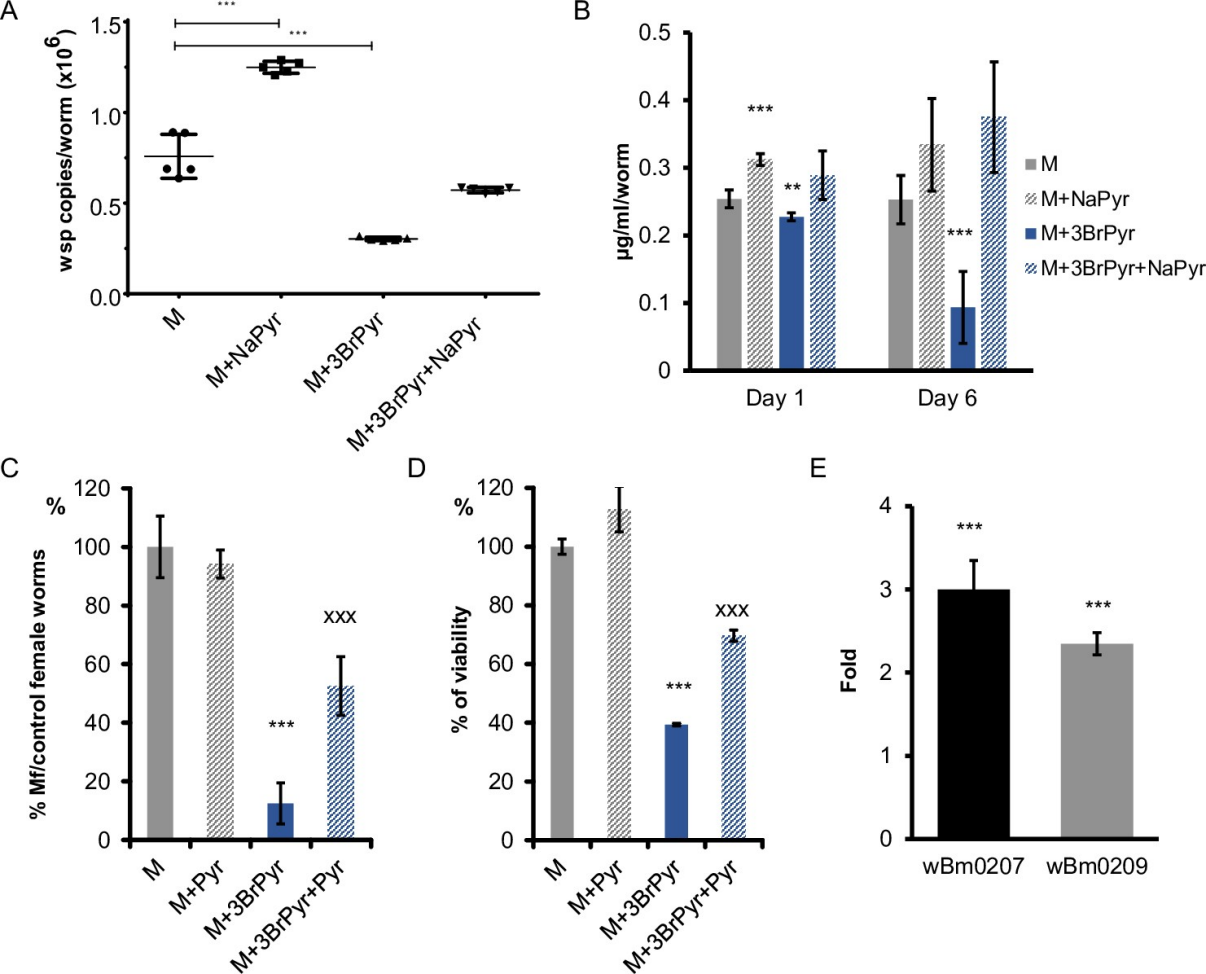
Conditional *in vitro* treatments of *B*. *pahangi* adult worms. A. The *wsp* copy number per worm in adult males after a 6-day treatment was measured by qPCR. The copy number reflects the abundance of bacteria in treated and control worms (***–p<0.001). B. Relative changes in the concentration of pyruvate within the worms measured after 1 and 6 days of treatment. Amount of pyruvate is expressed as μg per ml per worm. ***–p<0.001 as compared to relevant control (day 1 or day 6). C. Relative number of Mf released from treated females during the last 2 days of the 6-day treatment. Number of Mf released from control was assigned as 100%. ***–p<0.001 as compared to control and ^xxx^–p<0.001 as compared to M+3BrPyr group. D. Viability of females treated for 6 days. The viability of control worms was taken as 100%. ***–p<0.001 as compared to control and ^xxx^–p<0.001 as compared to M+3BrPyr group. E. Relative gene expression of wBm0209 and wBm0207 genes in treated worms. The analysis showed that treatment with sodium pyruvate induced the expression of bacterial genes as compared to control (***–p<0.001). M–medium control; M+NaPyr–treatment with sodium pyruvate; M+3BrPyr–treatment with 3BromoPyruvate; M+3BrPyr+NaPyr–treatment with 3BromoPyruvate and sodium pyruvate.

To study the effect of pyruvate on the *Wolbachia* population in the worms, adult *B*. *pahangi* were cultured for 6 days in complete medium supplemented with sodium pyruvate (12.5 mM) as an external source of pyruvate. *Wolbachia* load was determined in male rather than female worms to distinguish the effects of sodium pyruvate on *Wolbachia* within the hypodermal chord (the only location in males where they can be found) from those present within ovaries and embryos. After treatment with sodium pyruvate the *Wolbachia* population per male worm increased by almost 2-fold (p<0.001) compared to control male worms (**Fig 1A**).

To evaluate the concentration of pyruvate in treated worms, we used female worms for the assay because they are on average 2-times larger than male worms. Notably, the concentration of pyruvate rapidly and significantly increased (p<0.001) in the sodium pyruvate-treated female worms, with a significant difference as compared to untreated worms by day 1 of treatment but with no significant difference by day 6 of treatment (**Fig 1B**). Since the *Wolbachia* population significantly expanded in the sodium pyruvate-treated worms (**Fig 1A**) over the 6 days of the experiment, we hypothesize that the excess pyruvate was metabolized by the bacteria.

Filarial *Wolbachia* are embedded within the glycogen (polymeric form of glucose) stored in the hypodermal chord of the filarial host [8,21]. Recently, we have also shown that *w*Bm depends on both glycogen and glucose to persist in the *B*. *malayi* worms [21]. We hypothesized that the *Brugia* glycolysis pathway is a primary source of pyruvate for the bacteria that reside within the filarial host cell cytoplasm. To further investigate whether pyruvate, the end-product of glycolysis, is used by the bacteria and thus important for their fitness, we blocked the entire glycolysis pathway with 3-Bromopyruvate (3BrPyr). The *Wolbachia* population in adult males treated with 3BrPyr for 6 days decreased significantly by almost 3-fold (p<0.001) (**Fig 1A**). Moreover, the amount of pyruvate in the 1-day or 6-day treated female worms also significantly decreased as compared to the control samples (**Fig 1B**). Importantly, the inhibitory effect of 3BrPyr was reversed by adding external pyruvate to the medium. The combined treatment with sodium pyruvate and 3BrPyr restored both the population size of the bacteria within the adult male worms (**Fig 1A**) as well as the amount of pyruvate in the treated female worms (**Fig 1B**).

Treatment with external pyruvate did not significantly change the number of microfilariae released from the treated female worms compared to control worms (**Fig 1C**), nor did it significantly affect the viability of adult female worms (**Fig 1D**). However, blocking of glycolysis by treatment with 3BrPyr, which significantly reduced the number of *Wolbachia* (**Fig 1A**), also reduced both the number of Mf secreted by treated females by 80% and the viability of these females as compared to control female worms by 60% (**Fig 1C and 1D**). Both phenotypes—Mf production and viability of the worms—were partially but significantly (p<0.001) rescued by adding external sodium pyruvate to the 3BrPyr-treated worms (**Fig 1C and 1D**).

To confirm the co-dependence of *Wolbachia* and *Brugia* on the synthesis and use of pyruvate, we repeated the conditional culturing experiments described above with *A*. *viteae* (females), a *Wolbachia*-free rodent filarial nematode. Although treatment with pyruvate resulted in a significant increase of pyruvate concentration within the female adult worms as compared to the control, the inhibition of glycolysis by 3BrPyr (1 μM, the same concentration used for the treatment of *B*. *pahangi*) did not significantly affect the viability of the worms and/or the concentration of pyruvate within the treated *A*. *viteae* worms. However, the combined treatment of 3BrPyr and sodium pyruvate resulted in a significant increase in pyruvate concentration in the worms as compared to the control or 3BrPyr treatment alone (**S1 Fig**). These results indicate that in filarial worms that do not have *Wolbachia*, the external pyruvate accumulates in the treated worms without being efficiently metabolized.

### External pyruvate stimulates the expression of *Wolbachia* genes associated with the use of pyruvate

To better understand how pyruvate, either produced by the filarial host or introduced externally, regulates *Wolbachia* transcriptionally and thus metabolically, we measured the effects pyruvate had on the expression of two bacterial genes that encode enzymes known to use pyruvate as their substrate in a) gluconeogenesis, where wBm0209 converts pyruvate to phosphoenolpyruvate P-PEP—the first step in gluconeogenesis; and b) the TCA cycle, where wBm0207 performs the first reaction in converting pyruvate to Acetyl-CoA, which then enters the TCA cycle to produce energy. Female worms cultured in medium supplemented with sodium pyruvate had a 2.4- and 3.0-fold increase (p<0.001) in the expression levels of wBm0209 and wBm0207, respectively, as compared to control samples (**Fig 1E**). These results support the premise that pyruvate can be consumed by the bacterial gluconeogenesis pathway and/or the TCA cycle.

### Pyruvate kinase inhibitor III treatment affects *Wolbachia* and *Brugia* fitness

**Fig 2:**
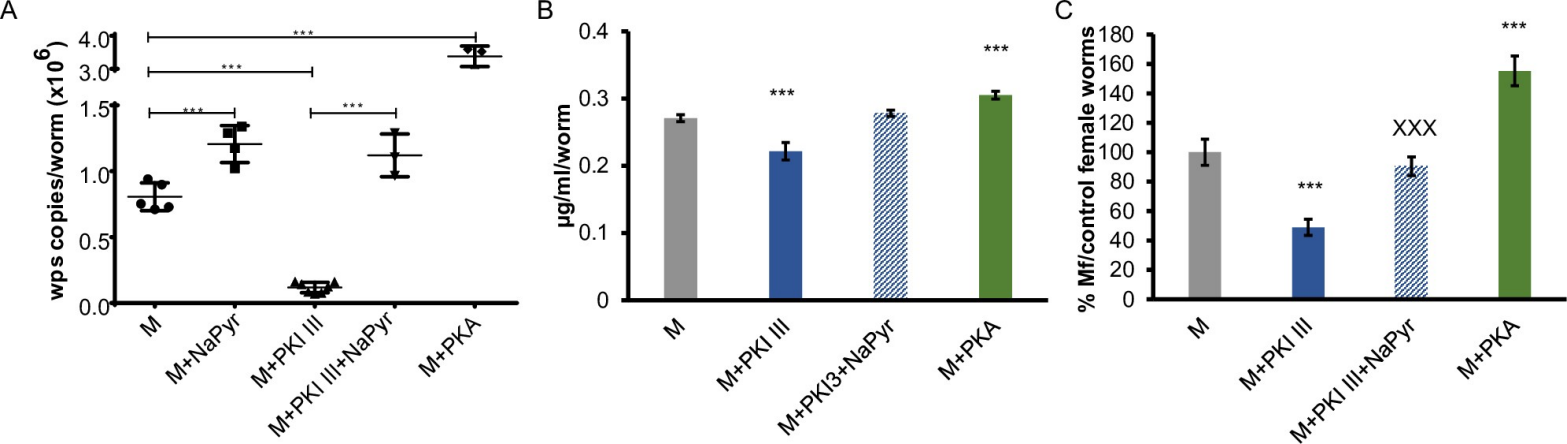
Effect of treatment with a pyruvate kinase inhibitor on the fitness of both *Wolbachia* and the worms. A. The *wsp* copy number per worm in adult males after a 6-day treatment was measured by qPCR (***–p<0.001). B. Relative changes in the concentration of pyruvate within the worms assigning 100% for control samples on day 1 of the treatment (***–p<0.001). C. Relative number of Mf released from female worms during the last 2 days of the 6-day treatment. Number of Mf released from control was assigned as 100%. ***–p<0.001 as compared to control and xxx–p<0.001 as compared to M+PKI III group. M–medium control; M+NaPyr–treatment with sodium pyruvate; M+PKI III–treatment with pyruvate kinase inhibitor III; M+PKI III+NaPyr–treatment with PKI III and sodium pyruvate; M+PKA–treatment with PK activator.

As inhibition of glycolysis by 3BrPyr had a significant impact on bacterial and worm survival as well as on Mf secretion, we tested whether pyruvate kinase, the enzyme that catalyzes the final step of glycolysis and produces pyruvate and ATP, could be an effective drug target. Using a similar set of experiments as the ones described above, we inhibited pyruvate kinase by adding a specific pyruvate kinase inhibitor (PKI III) into the culture medium in the absence or presence of external pyruvate. As previously observed, the addition of sodium pyruvate to male worms significantly increased the *Wolbachia* numbers (**Fig 1A and Fig 2A**). Importantly, inhibition of pyruvate kinase reduced the *Wolbachia* loads by 5-fold (p<0.001) as compared to the control worms. Moreover, treatment with PKI III also significantly reduced the amount of pyruvate within the treated female worms (36% reduction; p<0.001) (**Fig 2B**). The number of microfilariae released from female worms was also affected in treated worms, as females released at least two-fold fewer Mf than the control worms (**Fig 2C**).

Importantly, the effects of PKI III treatment were reversed by rescue experiments using media supplemented with external sodium pyruvate. The number of bacteria in the treated male worms was restored, as well as the concentration of pyruvate within the female worms and the ability of females to produce and release Mf *in vitro* (**Fig 2**). All the observed phenotypes were comparable to those seen in control samples.

We also tested whether a pyruvate kinase activator (PKA) would have a beneficial effect on bacterial growth. Treatment with PKA dramatically increased the *Wolbachia* loads in male worms as compared to controls (**Fig 2A**). These results further confirmed the importance of glycolysis and, in particular, the final step of glycolysis involving pyruvate kinase for survival and fitness of the bacteria symbiont and its effect on the filarial parasites.

### Filarial lactate dehydrogenase (LDH) may provide an alternative, but non-essential, source of pyruvate

Our previous studies established that a set of *B*. *malayi* glycolytic enzymes were associated with the *Wolbachia* surface protein (wBm0432) [20]. Our current results show that glycolysis is a primary metabolic pathway that provides its end-product pyruvate to both the worms and their symbiont, which explains functionally our previous observation. Interestingly, the previous studies also indicated that the *Brugia* lactate dehydrogenase (Bm-LDH, Bm3339) was complexed with the bacterial wBm0432 surface protein [20]. LDH catalyzes the conversion between lactate and pyruvate. We therefore tested whether LDH can provide *Wolbachia* with an alternative source of pyruvate and, consequently, regulate the physiological concentration of pyruvate near the surface of the bacteria.

**Fig 3:**
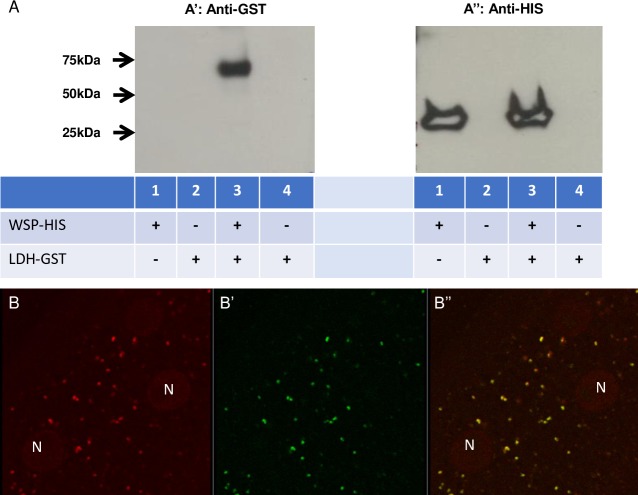
Association of Bm-LDH and wBm0432 of *Wolbachia*. A. Western blot of the pull-down assay confirming the formation of complexes between wBm0432-HIS and Bm-LDH-GST recombinant proteins. Lane 1, wBm0432-HIS immobilized on Ni-NTA beads (wBm0432-HIS-beads) was incubated with bacterial control extract (bacteria that did not express Bm-LDH-GST). Lane 2, free beads (no proteins were immobilized on the Ni-NTA beads) were incubated with bacterial control extract (bacteria did not express Bm-LDH-GST). Lane 3, wBm0432-HIS-beads were incubated with Bm-LDH-GST within the bacterial extract (bacteria expressing Bm-LDH-GST). Lane 4, free beads were incubated with Bm-LDH-GST within the bacterial extract (bacteria expressed Bm-LDH-GST). Western blot was treated with anti-GST (A’) or with anti-HIS (A”) antibodies. B, B’, B”. Confocal image showing the co-localization of *w*Bm (red, B) and LDH (green, B’) in the cytoplasm of the parasite’s lateral chord. B”. Merged red and green channels. Colocalization of red and green signals merged as yellow. Magnification 63x. Negative control is presented in **S2 Fig**.

We first confirmed using a pull-down assay that Bm-LDH is associated with the *Wolbachia* surface and, specifically, with the wBm0432 surface protein. The co-immunoprecipitation assay clearly established that recombinant protein Bm-LDH-GST can form stable complexes with the wBm0432-HIS protein (**Fig 3A**). This association within the worms was further confirmed by confocal microscopy analysis, which showed that Bm-LDH was co-localized with *Wolbachia* in the lateral chord of the *B*. *malayi* worms (**Fig 3B and S2 Fig**).

To further investigate whether LDH can provide an alternative source of pyruvate for the bacteria, LDH was inhibited by sodium oxalate (SO). As SO is a general inhibitor of LDH, we first tested the capacity of SO to inhibit filarial LDH using crude protein extracts prepared from adult filarial worms (*B*. *pahangi* and *A*. *viteae*) as compared to the human LDH using the LDH activity kit. Filarial crude protein extracts incubated with 1, 10 or 15 mM SO show a dose-dependent inhibitory effect of SO on the native nematode LDH with a similar pattern to that obtained with the human LDH (**S3 Fig**). The nematode LDH was, however, more sensitive to SO (25% inhibition in *Brugia* samples incubated at 1mM of SO vs. no effect on the human LDH and 70% inhibition in *Brugia* samples incubated at 10mM of SO vs. 19% inhibition of the human LDH). Data suggest that SO is a more potent inhibitor of the filarial LDH than of the human homologue. As this confirmed that SO was an effective inhibitor of *Brugia* LDH, its effect was then evaluated on *B*. *pahangi* adult worms *in vitro*.

**Fig 4:**
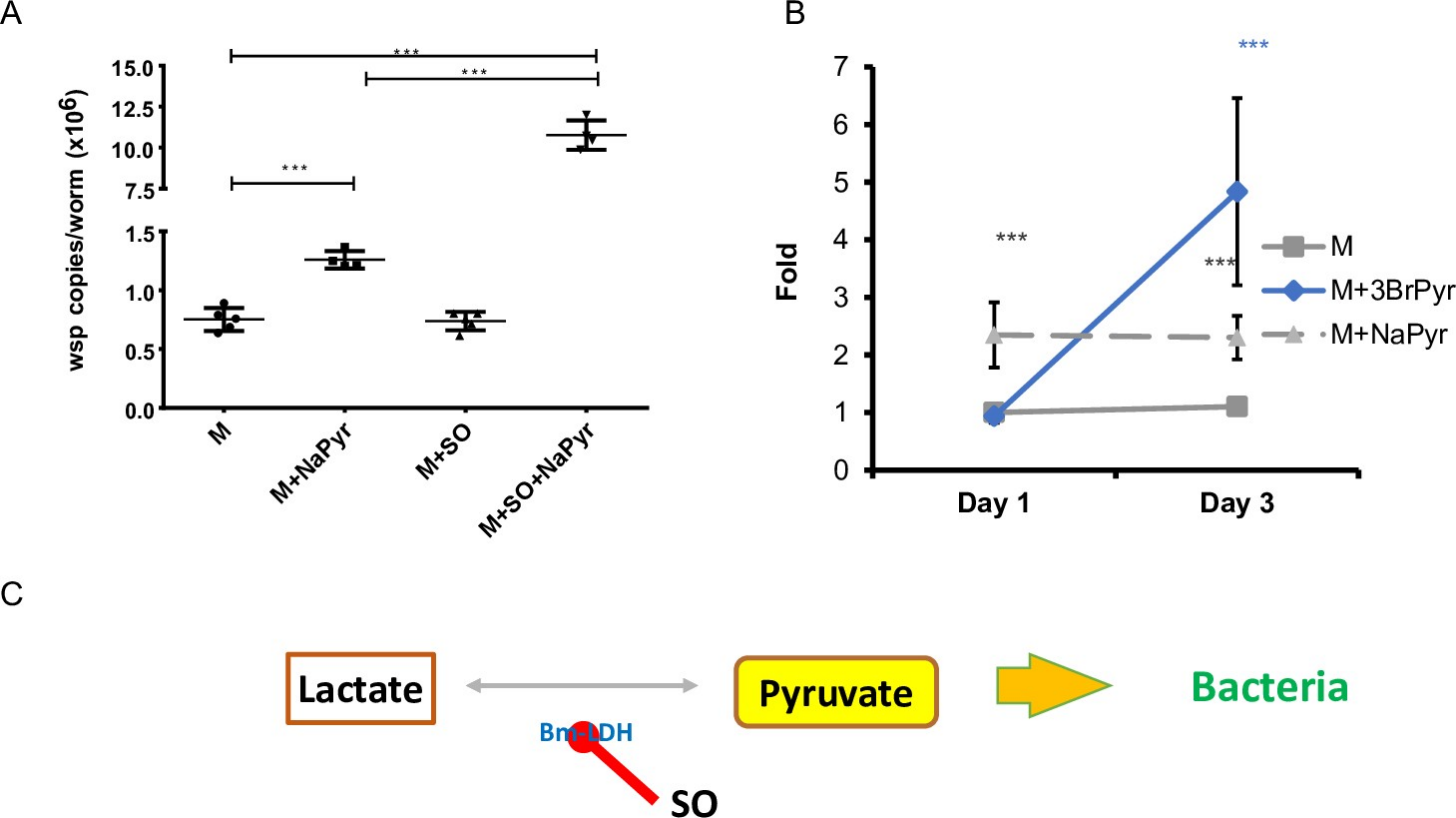
Effect of treatment with LDH inhibitor on the *Wolbachia*-parasite interaction. A. The *wsp* copy number per worm in adult males after a 6-day treatment was measured by qPCR (***–p<0.001). B. Relative gene expression analysis of *Bm-LDH* in samples treated with pyruvate (M+Pyr, grey, triangle), 3BromoPyruvate, inhibitor of glycolysis (M+3BrPyr, Blue, diamond) and control (M, gray, square). The analysis showed that pyruvate treatment induced expression of *Bm-LDH* after the first day of treatment. Inhibition of glycolysis only activated the expression of *Bm-LDH* in worms after 3 days of treatment. C. Schematic presentation of the role of Bm-LDH in pyruvate distribution between bacteria and conversion into lactate in parasite cells.

When *Brugia* male worms were cultured in the presence of 10 mM of SO for 6 days, inhibition of Bm-LDH did not significantly affect *Wolbachia* loads as compared to the control (**Fig 4A**). Notably, worms that were incubated with both SO and external pyruvate had 5-fold more bacteria (p<0.001) as compared to the control (complete medium), and 3-fold higher numbers (p<0.001) when compared to worms cultured in media supplemented with only pyruvate (**Fig 4A**).

To test whether lactate might be an alternative source of intracellular host pyruvate for the bacteria, particularly when glycolysis is inhibited, we analyzed the effect inhibition of glycolysis has on the expression levels of *Bm-LDH*. The expression of *Bm-LDH* did not change in worms in which glycolysis was inhibited by 3BrPyr for 1 day, but it was significantly overexpressed on day 3 of the treatment (**Fig 4B**). These results suggest that Bm-LDH may provide an alternative source of intracellular host pyruvate to the symbiont, but it could not totally rescue the detrimental effect of 3BrPyr. Interestingly, inhibition of Bm-LDH by SO in the presence of sodium pyruvate significantly expanded the bacterial population within the male worms (by 4-fold as compared to the control), which was greater than when the worms were exposed only to sodium pyruvate (2-fold increase only). We predict that Bm-LDH converts the extra pyruvate into lactate, and by this process it might regulate the amount of pyruvate used by *Wolbachia* (**Fig 4C**). Consequently, the expansion of the bacterial population in the host cytoplasm is more limited than when Bm-LDH is inhibited by SO, and all the external pyruvate can be used by the bacteria facilitating their growth (**Fig 4A**). In support of this hypothesis, the *Bm-LDH* gene was overexpressed in worms treated with external pyruvate on day 1 and day 3 (2.5 and 2.2-fold increase respectively, p<0.001) (**Fig 4B**).

### Stage-specific expression levels of parasite enzymes that function during glycolysis, gluconeogenesis, and energy metabolism predict symbiotic interactions

**Fig 5:**
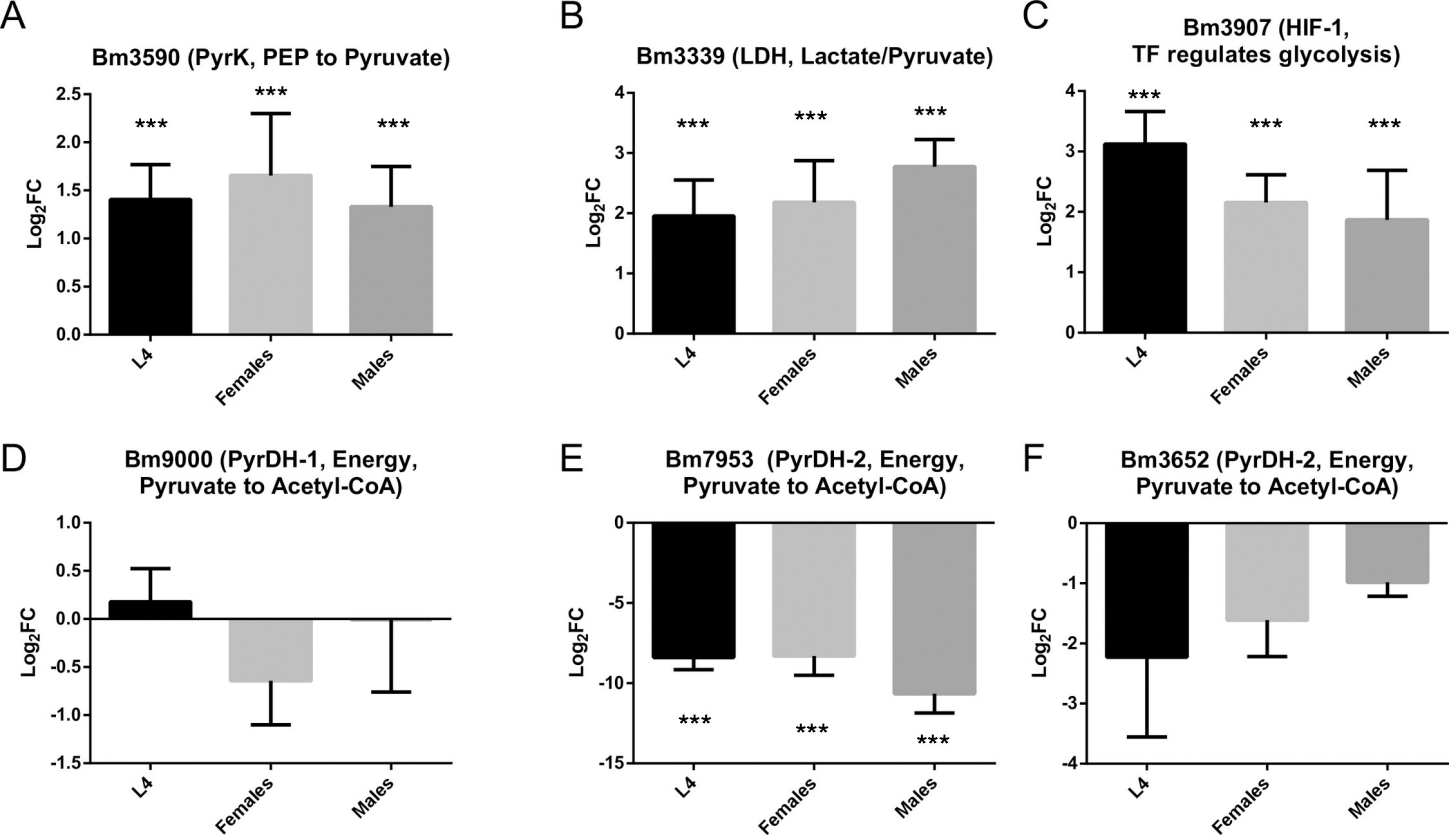
Analysis of transcriptomic data obtained from *B*. *malayi* L3, L4 and adult worms. A. Gene expression of *PyrK* (Bm3590) responsible for the final step in glycolysis (conversion of P-PEP to pyruvate). B. Gene expression of *LDH* (Bm3339) responsible for the conversion between lactate and pyruvate. C. Gene expression of *HIF-1* (Bm3907), transcriptional factor that initiates the expression of glycolytic enzymes. D, E, F. Gene expression of *PyrDH* (Bm9000 (D), Bm7953 (E), Bm3652 (F)) responsible for the conversion of pyruvate to Acetyl-CoA. ***–p<0.001 as compared to L3 samples.

Using published transcriptomic data, we analyzed glycolytic pathway gene expression in L4, a stage when the bacteria population is expanding dramatically, and in adult male and female worms [27] as compared to the infective L3 stage when the number of *Wolbachia* is at its lowest [24]. We first analyzed the expression of pyruvate kinase (PyrK), an enzyme that performs the last step in glycolysis converting phosphoenolpyruvate (P-PEP) to pyruvate in the worms [29]. This enzyme is not found in the *w*Bm or *w*Bp genomes. Therefore, if *Wolbachia* depends on pyruvate produced by the filarial glycolytic metabolic pathway, one would predict that the expression of the *PyrK* gene would increase when bacteria numbers are expanding in L4 and in adult worms. We observed a significant increase in the expression of *PyrK* (Bm3590) in L4 and adult worms as compared to L3 (**Fig 5A**). We also evaluated the regulation of glycolysis using the stage-specific *Brugia* transcriptomic data for the Hypoxia-inducible factor 1 (HIF-1), a heterodimeric transcription factor [30], and a major transcriptional activator of glycolytic genes [30,31]. Notably, *HIF-1* was significantly upregulated in L4 and adult worms as compared to L3 (**Fig 5C**).

We found that the expression of the *LDH* gene (Bm3339) was dramatically increased in L4 as compared to L3 and it remained high in adult worms (**Fig 5B**). While this enzyme can modulate the local concentration of pyruvate, how LDH regulates quantity and access to pyruvate requires further studies.

Finally, we analyzed the filarial pyruvate dehydrogenase (PyrDH), the first component enzyme of the pyruvate dehydrogenase complex that converts pyruvate to Acetyl-CoA and initiates the TCA cycle (*Wolbachia* ortholog, wBm0207 studied above, **Fig 1E**). According to the annotated database (wormbase.org), the genome of *B*. *malayi* encodes three *PyrDH* genes: Bm9000, Bm7953, and Bm3652. Comparative transcriptomic analysis has shown that the expression of Bm9000 is not significantly changed in L4 and adult worms when compared to L3. However, the transcript levels of Bm7953 and Bm3652 were significantly reduced in L4 and adult worms when compared to L3 (**Fig 5D–5F**). As *Wolbachia* increase the expression levels of their *PyrDH* gene (**Fig 1E**) during the development of mature stages of the worm, *Wolbachia* may also provide the filarial worms an alternative source of energy while the worms provide an intracellular source of pyruvate to the bacteria. This hypothesis will have to be further tested in future studies.

## Discussion

In many organisms, glycolysis is one of the most important metabolic pathways as it produces pyruvate and the high energy molecules ATP and NADH. Prokaryotic cells use pyruvate as one of their main sources for the production of energy and gluconeogenesis followed by nucleotide biosynthesis. In this study we investigated the role glycolysis and its end-product pyruvate play in the interactions between *Wolbachia* and its filarial host. While the genome of *Wolbachia*, an obligate symbiont of many filarial nematodes, does not encode several genes of the glycolytic pathway, it does encode all genes involved in gluconeogenesis [32]. Our previous studies have shown that to overcome this limitation, *Wolbachia* sequesters the parasite’s glycolytic enzymes fructose-bisphosphate aldolase, triosephosphate isomerase, enolase, glyceraldehyde-3-phosphate dehydrogenase and phosphoglycerate kinase as a complex onto its surface. We hypothesized that the glycolytic enzymes could provide a local supply of pyruvate to *Wolbachia* [20,21]. In this study, we have confirmed the importance of pyruvate for *Wolbachia* fitness by demonstrating that external pyruvate facilitates *Wolbachia* expansion within adult *Brugia*. We also showed that when glycolysis was inhibited using 3Br-pyruvate or Pyruvate kinase inhibitor III, the number of *Wolbachia* within the treated worms was significantly reduced, as well as the amount of pyruvate produced by the adult worms. These treatments also significantly reduced the release of microfilariae by the female adult worms and the viability of the adult females. Notably, the addition of external pyruvate to the 3Br-pyruvate or Pyruvate kinase inhibitor III treated worms reversed the effects that both compounds had on *Wolbachia* and on the worms. Interestingly, when worms were cultured with a pyruvate kinase activator, a compound that activates the last step of glycolysis and thus the production of pyruvate, the *Wolbachia* population in the adult parasites was also significantly increased. The outcomes of these studies further confirmed that glycolysis, and especially the final step of glycolysis involving the conversion of phosphoenolpyruvate (P-PEP) to pyruvate by pyruvate kinase, is essential for the symbiotic relationship between *Wolbachia* and its filarial host and for the fitness of both partners. Importantly, inhibiting glycolysis (3BrPyr) in *A*. *viteae*, a *Wolbachia*-free rodent filarial nematode, did not affect the viability of *A*. *viteae* worms. Collectively, it appears that pyruvate produced by the host can be used by *Wolbachia* to support a stable population within the cytoplasm of the filarial host. These results also clarify why *Wolbachia* would be sequestering host glycolytic enzymes onto its surface for its local consumption of the filarial synthesized pyruvate [20]. A similar mechanism has been described in mammalian tissues where enzymes of the glycolytic pathway assemble in complexes near the cell’s cytoskeleton, and their proximity with each other increases the speed of glucose breakdown [33,34]. It is important to mention that all the experiments performed in this study used the current optimized *in vitro* culturing condition for *Brugia* spp. adult worms of 5% CO_2_, which could have potential limitations as this condition may not be representative of the physiological O_2_ partial pressures within the mammalian host.

The importance of glycolysis and pyruvate was described in other members of the monophyletic lineage (Rickettsiales: Anaplasmataceae) containing the genera *Rickettsia*, *Anaplasma*, and *Ehrlichia*. *Wolbachia* is an alpha-proteobacterium and part of this lineage. All members of this clade share the defining characteristic of being obligate endosymbionts [35]. *Anaplasma phagocytophilum* is an emerging tick-borne pathogen in the United States, Europe, Africa, and Asia, with increasing numbers of infected people and animals every year [36]. *A*. *phagocytophilum* and *Rickettsia prowazekii* share 469 genes [37], and neither bacteria can use glucose as a carbon or energy source. *A*. *phagocytophilum* only has a partial glycolysis pathway with the first step missing and the pathway starting with fructose 1,6-biphosphate. However, both bacteria, like *Wolbachia*, have metabolic pathways that can use pyruvate in the following pathways: the metabolism of pyruvate, the tricarboxylic acid cycle (TCA, energy metabolism) [38], and gluconeogenesis (the first step in the process of purine/pyrimidine biosynthesis and *de novo* nucleotide biosynthesis). Furthermore, *Anaplasma* was also shown to manipulate the host’s metabolic pathway to increase its infection and transmission rates in both of its vertebrate and invertebrate (tick) hosts. In particular, transcriptomic and proteomic studies of tick cells infected with *A*. *phagocytophilum* revealed that the infection activated the host’s glycolytic pathway [36].

Regulation of glycolysis has also been studied in other intracellular bacteria-host interactions. Hypoxia-inducible factor 1 α (HIF-1), a heterodimeric transcription factor that transcriptionally activates glycolytic genes [31], was significantly upregulated in tick tissues and ISE6 cells in response to *A*. *phagocytophilum* infection [36]. Notably, *bma-HIF-1* was upregulated in L4 and adult worms as compared to L3 worms, stages at which *Wolbachia* population is expanded. This further suggests that worms with increased numbers of bacteria require higher expression of the enzymes that produce pyruvate.

In distant bacterial groups, the role of glycolysis in supporting intracellular survival of bacteria has also been reported. Infection with the intracellular pathogen, *Mycobacterium tuberculosis*, reduces the levels of glucose in mice [39]. The transcription of most glycolytic genes was enhanced after *Listeria monocytogenes* infection in mice [40]. All observations suggest that host-bacteria associations might be based on both transient and lasting metabolic cooperation. Establishing that glycolysis is one of the underlying essential mechanisms of symbiosis in filarial worms could have a broader impact on studies of these other intracellular bacteria-host interactions in which the role of glycolysis in supporting intracellular survival of bacteria was reported.

As mentioned above, the *Wolbachia* genome does not encode PK [32] and, therefore, the glycolytic pathway is not fully present, meaning bacteria must depend on the host’s glycolytic pathway, including PK, to produce pyruvate. Previous transcriptomic studies showed that the expression of *PK* (Bm3590, **Fig 5A**) was upregulated in L4 and adult worms as compared to L3, stages that are known to be accompanied by a highly increased bacterial population [24]. When the *Wolbachia* population was decreased by treatment with doxycycline, the transcript level of *Bm-PK* was negligible in the treated adult worms [41]. Such dependence between PK expression and *Wolbachia* numbers and its activity suggests that this enzyme plays a key role in the symbiosis. Therefore, we evaluated whether pyruvate kinase could be a promising indirect drug target for anti-*Wolbachia* and, consequently, anti-filariae treatment. Our results (**Fig 2**) clearly validated this premise as treatment with a PK inhibitor led to a significant reduction in the *Wolbachia* population (more than 2-fold) and affected the fitness of the treated parasites, as measured by the number of microfilariae released *in vitro* into the media. These findings further support future consideration of glycolysis as an alternative drug target for anti-symbiosis treatment. In a previous study, we have shown that *Brugia* aldolase-2 is also important for the survival of both *Wolbachia* and the parasite, as well as for worm reproduction [21]. Hence, future studies using *in vivo* filarial infection models could further substantiate whether the PK inhibitor (and/or other inhibitors of glycolysis) can become candidate drugs to treat human filarial parasites that harbor *Wolbachia*. There are a number of studies where PK was also considered as a potential drug target. These studies range from anti-parasite (trypanosome) to anti-cancer (human) treatments [42,43]. Human pyruvate kinase (PKM2) is considered as a potential target for anti-cancer treatment. The inhibition of glycolysis—an active process in cancer cells—by targeting PKM2 in melanoma cells with Lapachol led to decreased ATP levels and inhibition of cell proliferation [44,45]. The *Brugia* PK protein has 61% similarity with the human pyruvate kinase (PKM, NP_872270). We believe that development of selective drugs that target *Brugia* glycolytic enzymes (including PK) with minimum or no effect on human cells is feasible, however, this should be confirmed in future drug discovery and validation studies.

Our previous studies have shown that the *Brugia* lactate dehydrogenase is also associated with the bacterial surface protein (wBm0432) as part of the complex with the host glycolytic enzymes [20]. We therefore also addressed the possibility that an alternative pathway involving LDH may also regulate local intracellular concentration of pyruvate in proximity of *Wolbachia*, which are localized in the parasite’s hypodermal cells, and thus possibly also control bacterial growth and its mutualistic functions. In the present study, we first confirmed that wBm0432 and Bm-LDH can form a complex and are colocalized around *Wolbachia* (**Fig 3**). However, this pathway appears to not be essential for preserving or expanding the *Wolbachia* population as inhibition of Bm-LDH using SO did not affect the number of *Wolbachia* in the treated worms. Interestingly, when Bm-LDH was inhibited in the presence of external pyruvate, the combined treatments dramatically increased bacterial numbers, as compared to the control (4-fold more, p<0.001) or as compared to the treatment with external pyruvate only (2.5-fold more, p<0.001). This suggests that when worms are cultured with external pyruvate, the pyruvate can be used by both *Wolbachia* and the host’s LDH, but when LDH is inhibited, all available pyruvate can be consumed by the bacteria resulting in a greater number of bacteria within these treated adult worms. The *Bm-LDH* gene expression increased on day 1 and 3 after treatment with pyruvate, further confirming that pyruvate can be consumed not only by the bacteria, but that it can also be converted into lactate by Bm-LDH to further regulate the local concentration of this metabolite (**Fig 4C**). Moreover, when the transcript levels of *Bm-LDH* were analyzed during the development of L3 to L4, and from L4 to adult worms, stages of the parasite in which the population of *Wolbachia* per worm increases by more than 600-fold [24], we found that the levels of *Bm-LDH* increased more than 4-fold as compared to L3 (**Fig 5B**). Thus, it is still possible that Bm-LDH might have a more important role in symbiosis, but mostly during worm development.

Pyruvate is a key metabolite that sits at the intersection of the network of metabolic pathways and can be used in gluconeogenesis, energy metabolism, and amino acid (alanine) synthesis. Here we showed that exposure of *Brugia* adult worms to extra pyruvate treatment increased the expression levels of bacterial enzymes wBm0207 (ortholog of *PyrDH*) and wBm0209 that use pyruvate for the TCA cycle and gluconeogenesis, respectively. Interestingly, parasite transcript levels of *Bm-PyrDH* enzymes, which use pyruvate for the synthesis of Acetyl-CoA via the TCA cycle in the mitochondria, decreased during the development of worms in gerbils as compared to vector-derived L3 (**Fig 5D–5F**) and when the bacteria loads are known to be significantly expanded [24]. Similarly, infection of tick cells with *A*. *phagocytophilum* also results in reduced gluconeogenesis and the TCA cycle in tick midguts [36]. Our data, therefore, suggest that *Wolbachia*, like *Anaplasma*, may reduce TCA cycle activity in its host. When *Wolbachia* are eliminated using antibiotics, the expression of *PyrDH* was 2.67-fold higher in doxycycline-treated adult worms as compared to the control [41], suggesting that *Wolbachia* likely play an important role in balancing energy metabolism in the filarial worms.

## Supporting information

S1 Fig Conditional *in vitro* treatments of *A*. *viteae* adult worms. A. Relative changes in the concentration of pyruvate within the worms measured after 6 days of treatment. Amount of pyruvate is expressed as μg per ml per worm. ***–p<0.001 as compared to control. B. Viability of females treated for 6 days. The viability of control worms was taken as 100%. M–medium control; M+Pyr–treatment with sodium pyruvate; M+3BrPyr–treatment with 3BromoPyruvate; M+3BrPyr+Pyr–treatment with 3BromoPyruvate and sodium pyruvate.
(TIF)

S2 Fig Test of specificity of anti-LDH antibodies. A. Western blot of proteins expressed by bacteria with LDH-GST construct (1) and proteins extracted from *Brugia malayi* adult female worms (2) probed with anti-LDH antibodies. Expected size of LDH-GST is 62kDa and Bm-LDH– 36kDa. B. Confocal image of *Brugia malayi* adult female stained with secondary anti-rabbit antibodies (FITC, green, B) and propidium iodide (wBm, red, B’). B” merged B and B’. Magnification 63x
(TIF)

S3 Fig The activity of LDH in protein extracts prepared from filarial nematodes (*B*. *malayi*, Bm or *A*. *viteae*, Av) and in human-LDH (Hu-LDH) is inhibited by SO. (PDF)
