# Supplementary figures and images for: Pyruvate produced by Brugia spp. via glycolysis is essential for maintaining the mutualistic association between the parasite and its endosymbiont, Wolbachia
Source: PLoS Pathog. 2019 Sep 30;15(9):e1008085. doi: 10.1371/journal.ppat.1008085 (PMC6791551; doi:10.1371/journal.ppat.1008085)

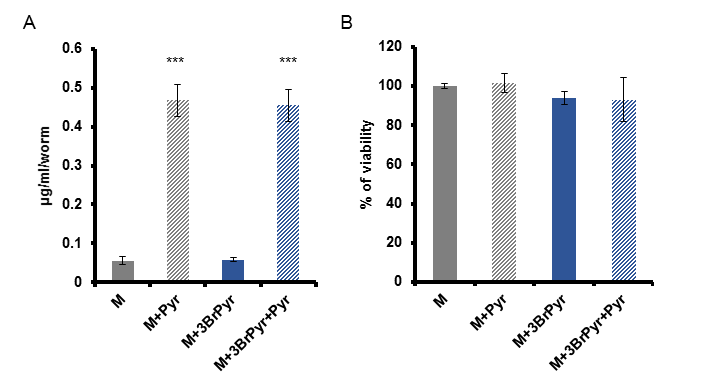

Supplement: S1 Fig — A. Relative changes in the concentration of pyruvate within the worms measured after 6 days of treatment. Amount of pyruvate is expressed as μg per ml per worm. ***–p<0.001 as compared to control. B. Viability of females treated for 6 days. The viability of control worms was taken as 100%. M–medium control; M+Pyr–treatment with sodium pyruvate; M+3BrPyr–treatment with 3BromoPyruvate; M+3BrPyr+Pyr–treatment with 3BromoPyruvate and sodium pyruvate. (TIF) [file ppat.1008085.s001.tif]

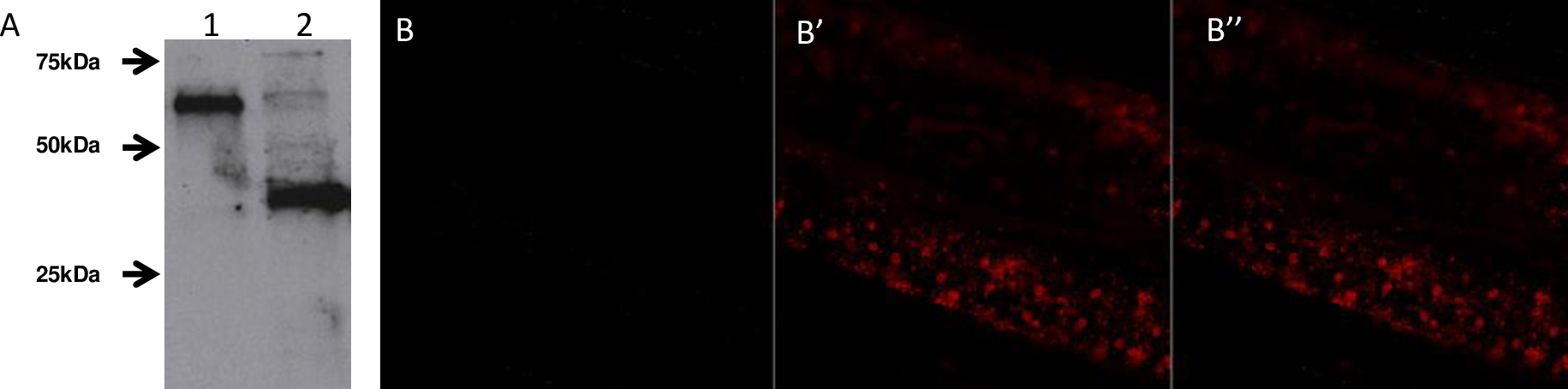

Supplement: S2 Fig — A. Western blot of proteins expressed by bacteria with LDH-GST construct (1) and proteins extracted from Brugia malayi adult female worms (2) probed with anti-LDH antibodies. Expected size of LDH-GST is 62kDa and Bm-LDH– 36kDa. B. Confocal image of Brugia malayi adult female stained with secondary anti-rabbit antibodies (FITC, green, B) and propidium iodide (wBm, red, B’). B” merged B and B’. Magnification 63x (TIF) [file ppat.1008085.s002.tif]

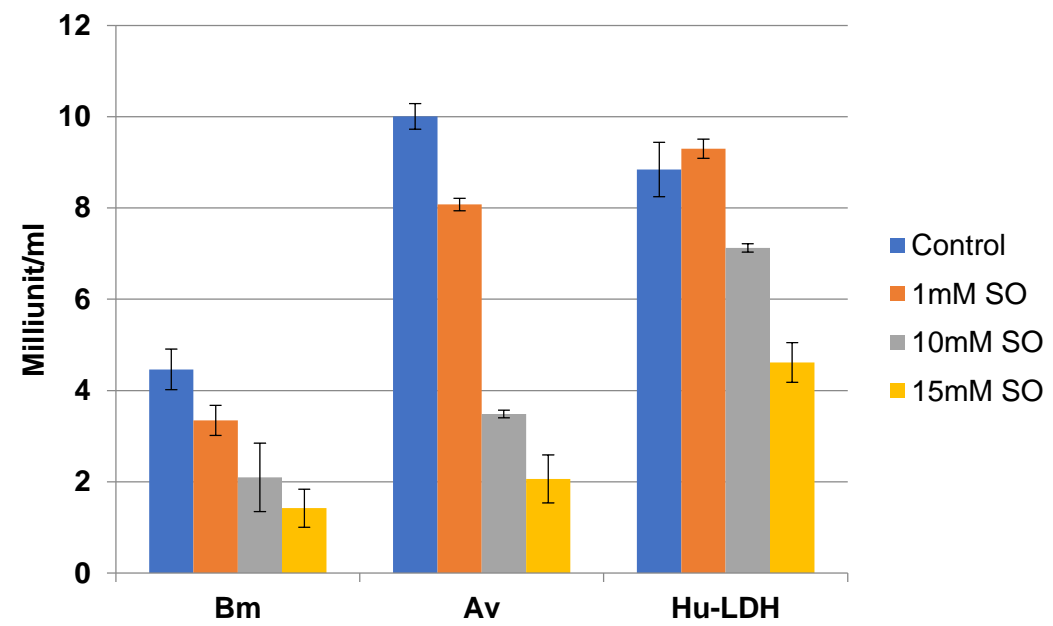

Supplement: S3 Fig — (PDF) [file ppat.1008085.s003.pdf]
